# Convergent Validity between Electromyographic Muscle Activity, Ultrasound Muscle Thickness and Dynamometric Force Measurement for Assessing Muscle

**DOI:** 10.3390/s23042030

**Published:** 2023-02-10

**Authors:** Umut Varol, Marcos J. Navarro-Santana, Juan Antonio Valera-Calero, Sergio Antón-Ramírez, Javier Álvaro-Martínez, María José Díaz-Arribas, César Fernández-de-las-Peñas, Gustavo Plaza-Manzano

**Affiliations:** 1 Escuela Internacional de Doctorado, Universidad Rey Juan Carlos, 28933 Alcorcón, Spain; 2 Department of Radiology, Rehabilitation and Physiotherapy, Faculty of Nursery, Physiotherapy and Podiatry, Complutense University of Madrid, 28040 Madrid, Spain; 3 Grupo InPhysio, Instituto de Investigación Sanitaria San Carlos (IdISSC), 28040 Madrid, Spain; 4 VALTRADOFI Research Group, Universidad Camilo José Cela, 28692 Villanueva de la Cañada, Spain; 5 Department of Physical Therapy, Occupational Therapy, Rehabilitation and Physical Medicine, Universidad Rey Juan Carlos, 28922 Alcorcón, Spain; 6 Cátedra Institucional en Docencia, Clínica e Investigación en Fisioterapia, Terapia Manual, Punción Seca y Ejercicio Terapéutico, Universidad Rey Juan Carlos, 28922 Alcorcón, Spain

**Keywords:** electromyography, ultrasound imaging, hand grip force, muscle fatigue, validity, sensors

## Abstract

Muscle fatigue is defined as a reversible decline in performance after intensive use, which largely recovers after a resting period. Surface electromyography (EMG), ultrasound imaging (US) and dynamometry are used to assess muscle activity, muscle morphology and isometric force capacity. This study aimed to assess the convergent validity between these three methods for assessing muscle fatigue during a manual prehension maximal voluntary isometric contraction (MVIC). A diagnostic accuracy study was conducted, enrolling 50 healthy participants for the measurement of simultaneous changes in muscle thickness, muscle activity and isometric force using EMG, US and a hand dynamometer, respectively, during a 15 s MVIC. An adjustment line and its variance (R^2^) were calculated. Muscle activity and thickness were comparable between genders (*p* > 0.05). However, men exhibited lower force holding capacity (*p* < 0.05). No side-to-side or dominance differences were found for any variable. Significant correlations were found for the EMG slope with US (r = 0.359; *p* < 0.01) and dynamometry (r = 0.305; *p* < 0.01) slopes and between dynamometry and US slopes (r = 0.227; *p* < 0.05). The sample of this study was characterized by comparable muscle activity and muscle thickness change between genders. In addition, fatigue slopes were not associated with demography or anthropometry. Our findings showed fair convergent associations between these methods, providing synergistic muscle fatigue information.

## 1. Introduction

Muscle contraction is produced by electric stimuli originating from the central nervous system (CNS) to motor neurons inserted into the muscle cells and branches. At this location, the electric stimulus produces the release of chemical neurotransmitters (acetylcholine) from synaptic vesicles to nicotinic receptors located on the motor endplate. This coupling induces muscle membrane depolarization, which is propagated to the sarcoplasmic reticulum to release the calcium ions needed for muscle contraction [1].

Although this physiological process is identical for all skeletal muscles, the endurance capacity for each muscle is not the same, as there are different muscle fibers described according to their properties and function [2]. Type I fibers contain several mitochondria and myoglobin, and are resilient and activated for sustained and prolonged submaximal demands (therefore, a high blood supply for this aerobic process is needed). In contrast, type II fibers provide more tension and more powerful forces during shorter times. As these are more anaerobic, less blood supply is needed (for this reason, they are also known as white muscle fibers) and they are characterized by rapid fatigue [3].

Muscle fatigue is defined as the reversible decline in performance after intensive use, which largely recovers after a resting period [4]. Chronic conditions including muscle atrophies (due to immobilization, sarcopenia or neurogenic etiology) or fiber type changes in chronic pain populations including neck pain [5] focused on muscle fatigue are a determinant factor affecting the functional capacity and quality of life of individuals [6].

Three methods widely used for assessing muscle performance are dynamometry, surface electromyography (EMG) and ultrasound imaging (US). These three methods can be used for monitoring changes in the applied force, electrical activity and muscle morphology, respectively, during a sustained maximum voluntary isometric contraction (MVIC) [7]. It should be noted that each method is characterized by its own strengths and limitations. For instance, US is portable, safe (as no ionizing radiation is used for acquiring the images), fast, accessible and provides real-time information, allowing the examination of immediate changes [8]. Due to these advantages and the range of imaging methods based on US (e.g., B-mode, M-mode, Doppler US, strain elastography, shear-wave elastography and panoramic US, among others [9]), it is a tool that is widely used in health sciences for the educational (i.e., teaching anatomy and pathologies [10]), research (i.e., to obtain objective measurements of muscle function, size, shape and composition [11]) and clinical settings (e.g., as a supportive tool for guiding needle interventions, visual feedback for motor control exercises or monitoring changes after interventions [12]). However, important limitations should be acknowledged. First, US is an operator-dependent method and certain exams require the examiner to be experienced so as to ensure good validity, reliability, sensibility and specificity [13]. Thus, due to the US interaction with the tissues (i.e., attenuation and refraction), some artifacts impede the visualization of some structures, including intra-articular elements or deep structures [14].

Regarding the strengths of EMG, its safety, ease and cost-effectivity should be highlighted. In contrast to needle EMG, it is not necessary to pierce the skin to record from single motor units to wide muscle areas [15]. This tool is not limited to providing the electrical activity difference between rest and contraction, facilitating cognition during motor control exercises [16], but also provides timing information, which is considered a determinant factor to differentiate clinical and asymptomatic populations [17]. The most important limitations of this tool are the limited ability to monitor muscle sites (as the neuromuscular system is complex, reducing the function to one or two channels may not represent the real function of the musculoskeletal system) [18], the possibility of “cross-talking” (a phenomenon where electrical activity from other muscles may bias the recording of the targeted muscle) and the need to normalize the data for correct interpretation [19].

Finally, hand dynamometers are characterized by their simplicity and portability for measuring the hand grip force and extrapolating the general strength level [20]. However, regular recalibrations are needed to ensure its accuracy and hand size is a determinant factor to be considered during the exams.

Despite previous research analyzing the association between US and EMG simultaneously with MVIC [21], evidence is limited regarding the convergent validity of these three methods for assessing simultaneously muscle fatigue during a sustained MVIC. Therefore, the aim of this study was to assess the association of the fatigue-adjusted line using US, EMG and dynamometry simultaneously during a sustained MVC to verify the association between isometric force capacity, electrical muscle activity and muscle morphology.

## 2. Materials and Methods

### 2.1. Study Design

This diagnostic accuracy study consisted of a single-group, cross-sectional concordance design. This study was conducted between October 2021 and February 2022 in a private university located in Madrid, Spain. Patients were evaluated once by two blinded examiners with +10 years of experience in each method. The study was conducted according to the directives of the Standards for Reporting of Diagnostic Accuracy Studies (STARD) [22] and the Enhancing the Quality and Transparency Of health Research (EQUATOR) guidelines and checklist [23]. All procedures were approved by a local Ethics Committee and conducted in accordance with the Declaration of Helsinki.

### 2.2. Participants

Healthy participants were recruited using fliers posted around the university campus. Participants had to be aged between 18 and 65 years and had to read and sign the informed consent form to be enrolled in the study. Exclusion criteria included (1) neurological, muscular, cardiovascular or any other condition contraindicating hand grip MVIC performance or contributing to pain appearance; (2) subjects with a body mass index (BMI) > 35 kg/m^2^ since this could bias the US and EMG measurements [21].

### 2.3. Sample Size Calculation

Sample size was calculated based on the only previous similar study analyzing simultaneously MVIC, US and EMG [21] (peak, but not fatigue) in healthy subjects. Accordingly, a minimum sample size of 38 participants could be considered appropriate. If this study was considered as a prognostic study, a range of 10 to 15 data points per potential predictor (with no more than five predictor variables) was recommended for avoiding overestimation of the results [24]. Therefore, considering the inclusion of 10 potential variables assessed in this study (excluding side and dominance as no differences are expected), the minimum sample size required was set at 100 measurements. Due to the cross-sectional nature of this study, no losses were considered.

### 2.4. Fatigue Measurement Instruments

**Figure 1:**
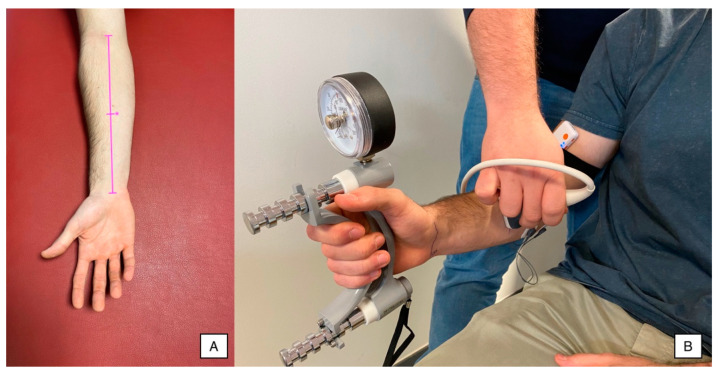
(**A**) Anthropometric references to identify the measurement point (*) and (**B**) EMG, US and hand dynamometry settings for simultaneous recording.

Data collection procedures were performed using a dynamometer, a surface EMG device and a US device simultaneously and synchronized during a sustained 15 s MVIC. A single measurement from both sides was acquired. Previously, participants were familiarized with the dynamometer and the starting position (subjects placed the Jamar in their hand, with the arm beside the trunk, the shoulder in a neutral position and the elbow flexed at 90°, and pulled the metal bar with their fingers), performing 2–3 submaximal contractions (to avoid early fatigue appearance), as illustrated in Figure 1. All procedures were conducted following the instructions described by Trinidad-Fernández et al. [21], as they demonstrated acceptable reliability.

#### 2.4.1. Ultrasound Imaging

All US procedures were conducted using the Alpinion Ecube i8 equipment (Gyeonggi-do, Republic of Korea) with a linear transducer E8-PB-L3-12T 3–12 MHz, setting the same parameters for all exams (frequency: 12 MHz; gain: 55 dB; dynamic range: 85 dB; brightness: 74), but the depth was adapted for each subject. Firstly, forearm length was determined as the distance from the elbow flexion crease to the wrist flexion crease [25]. The transducer was placed in the middle distance between these points, avoiding excessive pressure. In addition, this location was used to measure the forearm girth (Figure 1A). The transducer was glided medially to locate both the flexor digitorum superficialis and profundus and the cubital bone surface in the center of the image (Figure 1B).

#### 2.4.2. Hand Grip Force

The Hand Grip Force test is a simple, fast, reliable and relatively inexpensive test for assessing grip strength [26]. A Jamar hand dynamometer (JLW Instruments, Chicago, IL, USA) was used (Figure 1B). The handle diameter was set at 19.7% of the participant’s hand length, as recommended by Kong et al. [27]. One researcher explained and demonstrated the procedure before proceeding with the data collection. Participants were placed in the sitting position, holding the Jamar in their hand, with the arm beside the trunk, the shoulder in a neutral position and the elbow flexed at 90°. This procedure was conducted bilaterally, asking the participants to pull the metal bar with their fingers as hard as they could (respecting the positioning explained) for 15 s.

#### 2.4.3. Surface Electromyography

The surface electromyographic activity was collected using a mDurance Pro device (mDurance Solutions S.L., Granada, Spain). Data were transferred and processed using the mDurance software v.2.11.2 for Android 5.0. Prior to the electrodes’ placement, the skin was prepared following the recommendations provided by the Surface ElectroMyoGraphy for the Non-Invasive Assessment of Muscles [28]. Three 30-mm-diameter circular electrodes were used: one ground electrode was placed on the bone surface located at the medial humeral epicondyle and two reference electrodes were placed in line over the proximal third of the forearm to collect the muscle activity of the flexor digitorum superficialis and profundus as a single functional muscle (Figure 1B). This procedure followed the recommendations provided by the European Society of Electromyography regarding the spatial sensor characteristics and placement (reference electrodes located longitudinally to the fiber direction with an inter-electrode distance of 20 mm), electrode materials (Ag/AgCl) and signal conditioning (lower and upper cut-offs were set at 20 and 500 Hz, respectively, and sampling frequency was set at 1024 samples/second) and processing (root mean square, RMS) [29] based on previous studies assessing the same muscles during the same task [21,30,31].

### 2.5. Outcomes

#### 2.5.1. Isometric Force Holding

The Hand Grip Dynamometry provided the force (expressed in kilograms) during the 15 s MVIC hand grip test. Data for each second were collected and analyzed to measure force changes due to muscle fatigue.

#### 2.5.2. Muscle Thickness Change

**Figure 2:**
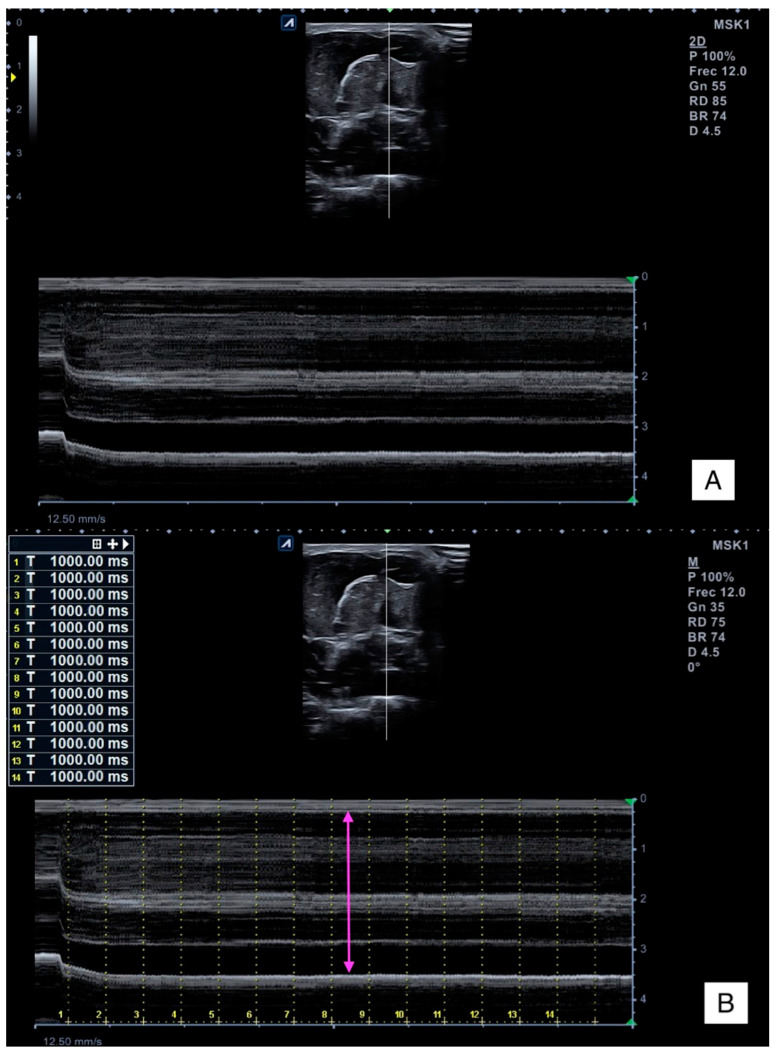
Raw M-mode ultrasound imaging (**A**) and thickness measurement (**B**).

M-mode ultrasound imaging provided information about the muscle thickness (*Y*-axis) per time (*X*-axis) (Figure 2A). Firstly, time frames of 1 s were marked in the M-mode images (where T_0_ corresponds to the contraction initiation). Then, muscle thickness was measured for each time point as the distance between the internal and superficial fascia of the flexor digitorum superficialis to the deep and internal fascia of the flexor digitorum profundi (Figure 2B).

#### 2.5.3. Muscle Activity Change

The surface EMG provided the muscle activity (expressing the amplitude in μV) during the 15 s test. Previous studies demonstrated the use of the flexor digitorum muscle during this task [32,33]. Data for each 0.25 s were collected and analyzed to measure muscle activity fatigue.

### 2.6. Data Analysis

**Figure 3:**
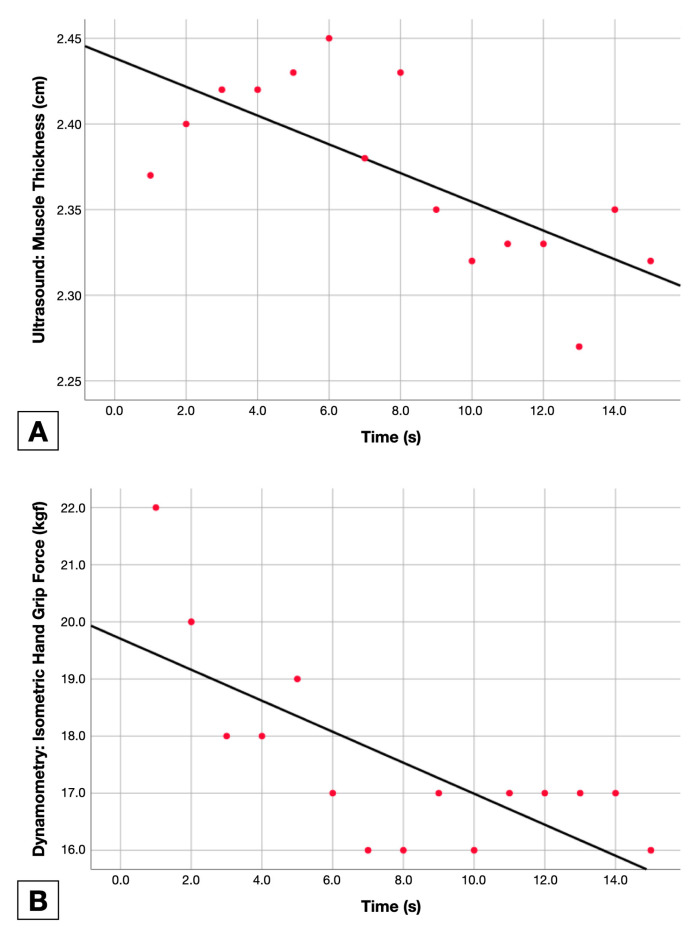

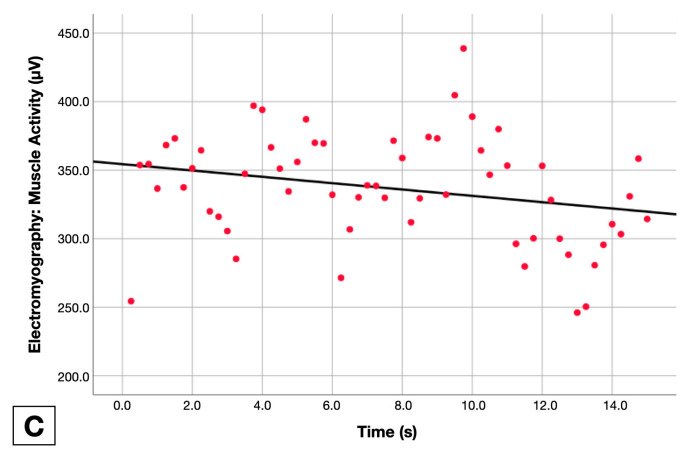
Fatigue measurement data points with adjusted line calculation using ultrasound imaging (**A**), Hand Grip Dynamometry (**B**) and electromyography (**C**).

The adjusted line for the function of each measurement method (force holding capacity, muscle activity and muscle thickness changes in the *Y*-axis) by time (*X*-axis) was calculated to obtain the linear regression slope and variance (R^2^). Adjusted lines for each method are available in Figure 3.

### 2.7. Statistical Analysis

All statistical analyses were conducted using the Statistical Package for the Social Sciences (SPSS) v.27 for Mac OS (Armonk, NY, USA). Normal distribution of each variable was verified using the Kolmogorov–Smirnov test if *p* > 0.05. Then, descriptive statistics were used to summarize continuous variables as the mean and standard deviation (if normally distributed) or median and interquartile range (if non-normally distributed) and categorical variables as frequency and percentage.

Student’s *t*-tests for independent samples were used to identify differences in gender (demographic, anthropometric and fatigue variables), side and dominance (both anthropometric and fatigue variables).

Pearson’s correlation coefficients (r) were used to calculate a multivariate correlation matrix including all demographic, anthropometric and fatigue variables. Association strength was interpreted accordingly with the values obtained (0–0.3 were poor, 0.3–0.5 fairly, 0.5–0.7 moderate and 0.8–1.0 strong), and direction (for continuous variables) was interpreted depending on the r sign (negative values as indirect correlations and positive values as positive correlations) [34].

## 3. Results

From a total of 52 volunteers responding to the announcements, two participants were excluded due to exceeding the BMI considered for this study. All registered measurements (n = 100) from the 50 participants enrolled were included in the analyses.

Table 1 and Table 2 summarize all demographic and anthropometric data of the total sample and divided into groups (gender, left/right sides and side dominance). Although participants had a comparable age (*p* > 0.05), men showed a greater BMI (difference = 0.6 to 4.7 kg/m^2^; *p* < 0.05) and in general were taller and heavier (difference = 0.08 to 0.16 m; *p* < 0.001). Forearm length and girth were larger in men compared to women (difference = 1.1 to 3.0 and 2.0 to 4.2, respectively; both *p* < 0.001). However, no side-to-side or dominant–non-dominant statistically significant differences were found (*p* > 0.05).

Table 3, Table 4 and Table 5 show the slope decrease rate and variance for each instrument by gender, side and dominance, respectively. For instance, the muscle activity assessed with EMG in males decreased at a rate of 1.08 μV per second. Therefore, after the 15 s maneuver, a mean decrease of 16.2 μV was observed. Fatigability results obtained from EMG, US and dynamometry were compared by gender, dominance and side. No linear regression slope differences were found between males and females for fatigue properties measured with EMG or US (*p* = 0.508 and *p* = 0.693, respectively). However, females were more capable of holding the MVIC compared with males (*p* = 0.022), demonstrating lower fatigability. On the other hand, comparisons between the right and left hand and between dominant and non-dominant sides showed no significant differences for EMG, US and dynamometry slopes (all *p* < 0.05).

Table 6 contains the correlation matrix. In general, we found anthropometric characteristics to be associated with several demographic features. Forearm girth was associated with age, height, weight, BMI, forearm length and gender (all *p* < 0.01), and forearm length was associated with height, weight, BMI (all *p* < 0.01) and gender (*p* < 0.05). Muscle activity fatigue and thickness change during the MVIC showed no significant correlations with demography or anthropometry (all *p* > 0.05). However, isometric force holding capacity was negatively associated with height (*p* < 0.05), forearm girth (*p* < 0.01) and length (*p* < 0.05). In addition, male sex was associated with greater force fatigue (*p* < 0.05). Finally, significant correlations between all methods used for quantifying muscle fatigue were found (*p* < 0.05).

## 4. Discussion

This study aimed to analyze the convergent validity of muscle activity assessed with EMG, muscle thickness assessed with US and isometric prehensile force assessed with dynamometry for measuring muscle fatigue. To our knowledge, this is the first study using adjusted line slopes with these tools to compare the association between them for measuring fatigue indicators such as electrical activity, muscle thickness and force capacity. Our findings showed significant weak associations between all methods, suggesting the use of all of them to provide synergistic information in the fatigue assessment, as it seems to be sensitive to different aspects and overcomes the limitations introduced previously (e.g., use of US for evaluating deep muscles not accessible with EMG). These findings could assist both clinicians and researchers to acquire overall information. The rationale for selecting the forearm muscles was supported by the anatomical disposition, which allows the simultaneous assessment with EMG, US and dynamometry and highlights the clinical importance of this muscular group [35]. Previous studies highlighted the importance of hand grip force as it is associated with a greater impact of rheumatoid arthritis [36], fibromyalgia [20], sarcopenia [37], cancer [38] or diabetes [39]. Additionally, it is considered an important predictor of mortality associated with falls [40,41] and heart failure [42].

Ultrasound imaging is widely used for assessing muscle morphology, histology and function [43,44]. Previous research demonstrated this tool to be useful in fatigue assessment to provide strategies aiming to alleviate muscle [45] or tendon failure [46] based on echogenicity and strain features, or as a discriminative and reliable tool for identifying clinical populations based on their ability to maintain an isometric contraction [47]. In addition, M-mode US could be a potential tool to be used in rehabilitative exercise programs, providing more effective feedback than tactile, with or without verbal advisements, in both exercise performance and retention success [48].

Shi et al. [49] conducted a sonographic study analyzing the feasibility of the muscle thickness change in measuring muscle fatigue while monitoring muscle activity during biceps brachii isometric contractions (80% MVIC) in eight healthy participants. Although they demonstrated this muscle deformation to be valid for measuring muscle fatigue, the sample size was small, and the procedures used for assessing the muscle thickness change with B-mode US were complex and not readily applicable during the clinical practice (e.g., software was needed to display results frame by frame, requiring off-line assessments). M-mode is an easy-to-use, accessible and applicable imaging mode, allowing both the examiner and participant to obtain real-time feedback in measuring the muscle thickness changes [50] of individual muscles, even if they are overlapped. Despite these methodological procedures’ differences, our results were consistent.

In this study, we focused on the Hand Grip Force as it is a widely considered outcome in a wide range of populations with different ages, sexes, states of health and pathological conditions [51]. One possible reason may be that muscle weakness is a risk factor in developing disability and dependence during activities of daily life, frailty and mortality [52]. Although Trinidad-Fernández et al. [21] found moderate and strong associations between EMG and muscle thickness, this association was found for the maximum grip force peak with the EMG score (calculated as the difference between the maximum and minimum peaks). Differences in the strength of the correlations between the two methodologies may be explained by the activity demands in muscle fibers derived from the cross-sectional and longitudinal designs.

Finally, previous studies evaluated electromyographic changes occurring during sustained muscle contractions for assessing muscle endurance [53,54,55]. For instance, Falla et al. [53] reported existing slope differences between healthy subjects and neck pain patients at 25% and 50% of MVIC for the sternocleidomastoid and anterior scalene muscles, suggesting the greater presence of type II fibers and greater fatigability of superficial neck muscles in neck pain patients compared with healthy subjects. In addition, Robinson et al. [54] and Elfving et al. [55] demonstrated significant differences for fatigue slopes between patients with chronic low back pain and asymptomatic subjects.

### Limitations

Although this study addressed many limitations of previous studies, several limitations remain. Firstly, this was a diagnostic accuracy study, and we limited this research to the agreement between methods and did not consider the fatigue demeanor. Further studies are needed assessing EMG, US and dynamometry differences between healthy and clinical populations for a better overall understanding of fatigue mechanisms. Secondly, we assessed both flexor digitorum muscles as a single unit, as we used surface EMG. Further studies should clarify whether there are muscle activity and thickness interactions between overlapped or adjacent muscles. Finally, fatigue assessments considering many other situations (e.g., populations, muscles, methods, contraction types and normalizations) would be of utility.

## 5. Conclusions

Our findings showed fair convergent associations between muscle thickness changes assessed with M-mode US, muscle activity changes assessed with surface EMG and hand grip force sustainment assessed with dynamometry. Although we found comparable muscle activity and muscle thickness changes between genders, men exhibited a greater decrease in force holding capacity. Fatigue slopes assessed with all methods were not associated with demography or anthropometry, but forearm girth and length were associated with force holding capacity. These findings suggest that these three methods assess different aspects of muscle fatigue, providing synergistic information about muscle activity, thickness and force capacity and allowing alternatives to overcome the limitations described previously for each method.

## Figures and Tables

**Table 1:** Participants’ demographic and anthropometric characteristics by gender.

Variables	Total Sample (n = 50)	Gender	
		Male (n = 26)	Female (n = 24)
Age (years)	22.3 ± 5.4	23.7 ± 6.7	20.8 ± 2.9
Height (m)	1.72 ± 0.09	1.78 ± 0.07	1.65 ± 0.06
Weight (kg)	70.8 ± 17.3	79.7 ± 18.3	61.1 ± 9.5
BMI (kg/m^2^)	23.5 ± 3.8	24.7 ± 4.2	22.1 ± 2.7
Forearm Length (cm)	25.6 ± 1.9	26.6 ± 1.6	24.5 ± 1.6
Forearm Perimeter (cm)	21.9 ± 2.4	23.4 ± 1.9	20.3 ± 1.9
Difference			
Age (years)		2.9 (−0.1; 5.9) *p* = 0.057	
Height (m)		0.12 (0.08; 0.16) *p* < 0.001	
Weight (kg)		18.6 (10.1; 27.0) *p* < 0.001	
BMI (kg/m^2^)		2.6 (0.6; 4.7) *p* = 0.013	
Forearm Length (cm)		2.0 (1.1; 3.0) *p* < 0.001	
Forearm Perimeter (cm)		3.1 (2.0; 4.2) *p* < 0.001	

Baseline values are expressed as mean ± standard deviation; differences are expressed as mean (95% confidence interval).

**Table 2:** Forearm anthropometric characteristic analyses by side and dominance.

Variables	Side		Dominance	
	Left (n = 50)	Right (n = 50)	Dominant (n = 50)	Non-Dominant (n = 50)
Forearm Length (cm)	25.6 ± 1.9	25.6 ± 1.9	25.6 ± 2.0	25.6 ± 1.9
Forearm Perimeter (cm)	21.6 ± 2.4	22.2 ± 2.4	22.2 ± 2.5	21.6 ± 2.4
Difference				
Forearm Length (cm)	0.0 (−0.7; 0.8) *p* = 0.927		0.0 (−0.7; 0.7) *p* = 0.967	
Forearm Perimeter (cm)	0.7 (−0.3; 1.6) *p* = 0.189		0.6 (−0.4; 1.6) *p* = 0.218	

Baseline values are expressed as mean ± standard deviation; differences are expressed as mean (95% confidence interval).

**Table 3:** Fatigue assessments with EMG, US and dynamometry by gender.

Variables	Gender	
	Male (n = 26)	Female (n = 24)
*Muscle Activity Changes: Surface Electromyography*		
Adjusted Line Slope	−1.08 ± 3.33	−0.70 ± 2.19
Difference	−0.38 (−1.52; 0.76); *p* = 0.508	
Variance (R^2^)	0.29 ± 0.26	0.30 ± 0.26
Difference	−0.006 (−0.11; 0.10); *p* = 0.910	
*Muscle Thickness Changes: Ultrasound Imaging*		
Adjusted Line Slope	−0.0043 ± 0.0110	−0.0036 ± 0.0062
Difference	−0.00074 (−0.0048; 0.00299) *p* = 0.693	
Variance (R^2^)	0.37 ± 0.30	0.34 ± 0.27
Difference	0.028 (−0.093; 0.149) *p* = 0.644	
*Force Holding Capacity: Hand Grip Dynamometry*		
Adjusted Line Slope	−1.39 ± 1.04	−1.04 ± 0.59
Difference	−0.35 (−0.69; −0.009); *p* = 0.022	
Variance (R^2^)	0.74 ± 0.21	0.76 ± 0.18
Difference	−0.018 (−0.099; 0.063); *p* = 0.656	

**Table 4:** Fatigue assessments with EMG, US and dynamometry by side.

Variables	Side	
	Left (n = 50)	Right (n = 50)
*Muscle Activity Changes: Surface Electromyography*		
Adjusted Line Slope	−0.50 ± 2.42	−1.27 ± 3.15
Difference	−0.648 (−1.78; 0.48); *p* = 0.258	
Variance (R^2^)	0.26 ± 0.25	0.33 ± 0.27
Difference	0.075 (−0.029; 0.181); *p* = 0.159	
*Muscle Thickness Changes: Ultrasound Imaging*		
Adjusted Line Slope	−0.0033 ± 0.0079	−0.0045 ± 0.0098
Difference	−0.000863 (−0.004598; 0.002872); *p* = 0.647	
Variance (R^2^)	0.38 ± 0.29	0.33 ± 0.29
Difference	0.273 (−0.188; 0.054); *p* = 0.272	
*Force Holding Capacity: Hand Grip Dynamometry*		
Adjusted Line Slope	−1.22 ± 0.80	−1.21 ± 0.93
Difference	0.0173 (−0.3327; 0.3673); *p* = 0.922	
Variance (R^2^)	0.75 ± 0.19	0.76 ± 0.20
Difference	−0.0078 (−0.0886; 0.0728); *p* = 0.847	

**Table 5:** Fatigue assessments with EMG, US and dynamometry by dominance.

Variables	Dominance	
	Dominant (n = 50)	Non-Dominant (n = 50)
*Muscle Activity Changes: Surface Electromyography*		
Adjusted Line Slope	1.21 ± 3.19	−0.57 ± 2.38
Difference	−0.769 (−1.897; 0.358); *p* = 0.179	
Variance (R^2^)	0.33 ± 0.27	0.26 ± 0.25
Difference	0.071 (−0.033; 0.177); *p* = 0.180	
*Muscle Thickness Changes: Ultrasound Imaging*		
Adjusted Line Slope	−0.0044 ± 0.0099	−0.0035 ± 0.0079
Difference	−0.0012 (−0.0049; 0.0025); *p* = 0.524	
Variance (R^2^)	0.32 ± 0.29	0.39 ± 0.28
Difference	−0.052 (−0.174; 0.068); *p* = 0.329	
*Force Holding Capacity: Hand Grip Dynamometry*		
Adjusted Line Slope	−1.21 ± 0.94	−1.22 ± 0.79
Difference	0.006 (−0.344; 0.355); *p* = 0.974	
Variance (R^2^)	0.75 ± 0.21	0.76 ± 0.18
Difference	0.0076 (−0.073; 0.088); *p* = 0.852	

**Table 6:** Pearson product moment correlation matrix.

Variables	1	2	3	4	5	6	7	8	9
1. Age									
2. Height	n.s.								
3. Weight	n.s.	0.758 **							
4. BMI	n.s.	0.455 **	0.919 **						
5. Forearm girth	0.295 *	0.577 **	0.663 **	0.600 **					
6. Forearm length	n.s.	0.811 **	0.473 **	n.s.	0.462 **				
7. Gender	n.s.	−0.678 **	−0.539 **	−0.350 *	−0.619 **	−0.546 **			
8. EMG slope	n.s.	n.s.	n.s.	n.s.	n.s.	n.s.	n.s.		
9. US slope	n.s.	n.s.	n.s.	n.s.	n.s.	n.s.	n.s.	0.359 **	
10. Dynamometry slope	n.s.	−0.337 *	n.s.	n.s.	−0.248 *	−0.278 **	0.202 *	0.305 **	0.227 *

* *p* < 0.05; ** *p* < 0.01.

## Data Availability

Not applicable.
